# The epidemiology of aggressive pituitary tumors (and its challenges)

**DOI:** 10.1007/s11154-020-09556-7

**Published:** 2020-05-02

**Authors:** Olaf M. Dekkers, Niki Karavitaki, Alberto M. Pereira

**Affiliations:** 1 Department of Clinical Epidemiology, Leiden University Medical Center, Leiden, Netherlands; 2 Department of Endocrinology, Leiden University Medical Center, Leiden, Netherlands; 3 Department of Clinical Epidemiology, Aarhus University Hospital, Aarhus, Denmark; 4 Institute of Metabolism and Systems Research, College of Medical and Dental Sciences, University of Birmingham, Birmingham, UK; 5 Centre for Endocrinology, Diabetes and Metabolism, Birmingham Health Partners, Birmingham, UK; 6 Department of Endocrinology, Queen Elizabeth Hospital, University Hospitals Birmingham NHS Foundation Trust, Birmingham, UK; 7 Center for Endocrine Tumors, Leiden University Medical Center, Leiden, Netherlands

**Keywords:** Aggressive pituitary adenoma, Carcinoma, Epidemiology, Incidence

## Abstract

Pituitary tumors are not rare if prevalence rates from autopsy or radiological series are considered; approximately 0.5% of all pituitary adenomas will come to medical attention. Less than 0.1% of these pituitary adenomas will become malignant, and probably around 0.5% of all detected adenomas will display an aggressive course. However, the exact incidence of both aggressive pituitary adenomas and pituitary carcinomas is unknown, as most data come from series with selected patients, such as surgically treated patients, which is likely not a reflection of all patients with a pituitary adenoma. An aggressive pituitary adenoma is not well-defined; even though an overarching definition, capturing both immunohistochemical and clinical characteristics is probably not waterproof, adoption of a widely accepted definition will be very helpful to harmonize research and establish more reliable epidemiological data.

Detailed knowledge on the epidemiology of a condition based on scientific literature, i.e. knowledge regarding its occurrence and prevalence, assumes a definition of the condition under study. Unfortunately, such common definition is lacking for aggressive pituitary tumors [1, 2], which hampers the clear epidemiological picture. The case is somewhat clearer for pituitary carcinomas, which are defined as pituitary tumors that exhibit metastasis [3].

Pituitary tumors are not rare if prevalence rates from autopsy or radiological series are considered: a meta-analysis from pooled autopsy and radiological series showed average prevalence rates of 14.4% (range 1–35%) and 22.5% (range 1–40%), respectively [4]. Most pituitary tumors thus go unnoticed during life.

The incidence of clinically apparent pituitary adenomas (quantifying the number of new cases), depends on sex and age. Based on data from registers with national coverage, prolactinomas are found most frequently, with an estimated incidence rate of 10/100,000 person-years in women of reproductive age [5]. The incidence for other adenomas is lower [5], being around 1–4/100,000 person-years for nonfunctioning adenomas, 0.4/100,000 person-years for GH secreting adenomas and 0.2/100,000 person-years for ACTH secreting adenomas. The prevalence of clinically significant adenomas that present to medical attention, as determined in cross-sectional community-based studies in Belgium and the UK ranged from 78 to 94 cases per 100,000 inhabitants [6, 7]. Combining these data with those from autopsy series as described above, suggests that approximately 0.5% of all pituitary tumors will come to medical attention, underlining the apparent very indolent nature of pituitary adenomas (see Fig. 1).

**Fig. 1:**
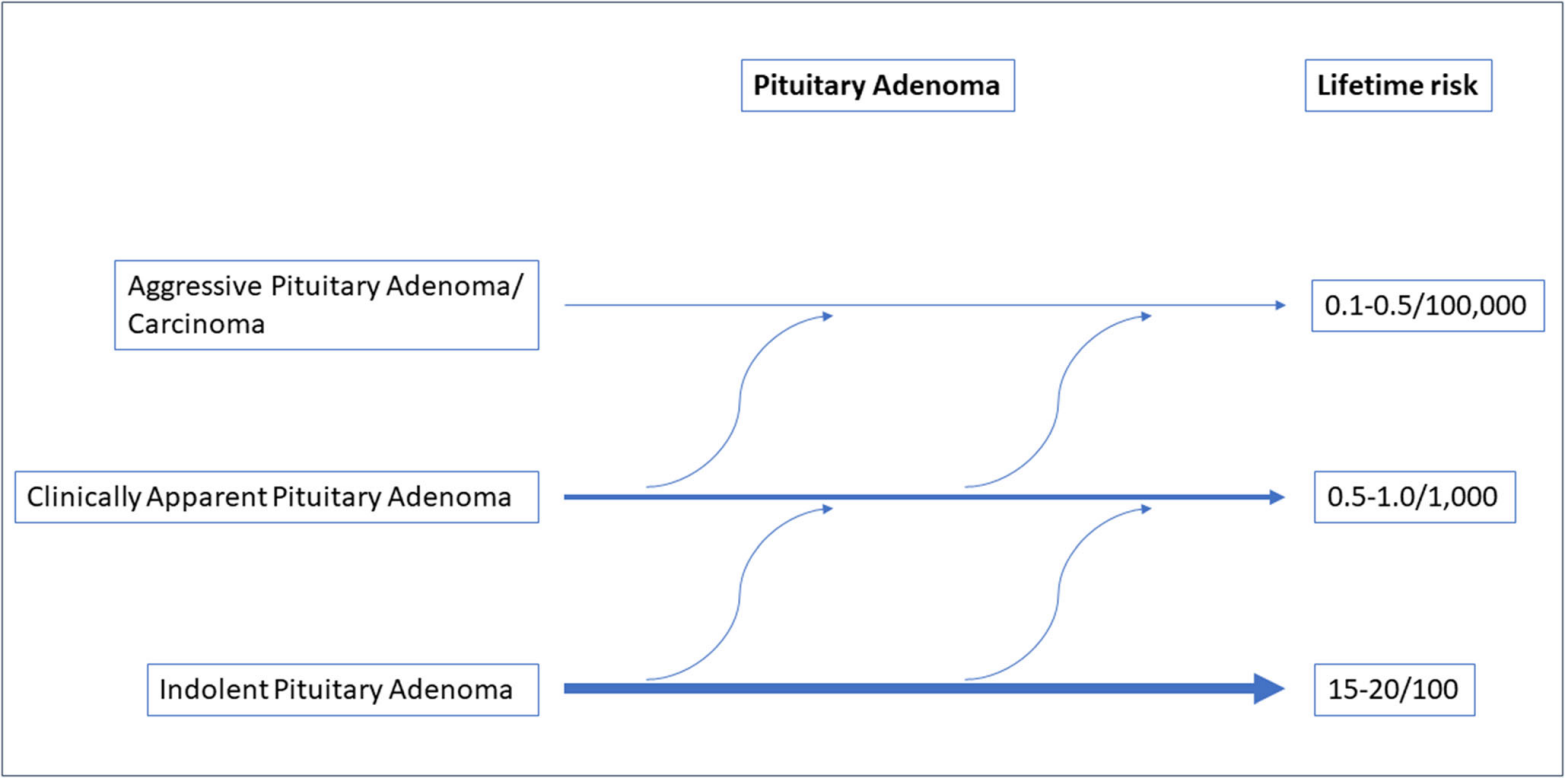
Estimated incidence for aggressive pituitary adenomas

Depending on size, all pituitary adenomas can show growth potential; in series with conservatively managed nonfunctioning adenomas (i.e. under monitoring surveillance), 10% of microadenomas and 23% of macroadenomas display growth [8]. Also, a decrease in tumor size is described [8, 9], confirming that there is no invariable growth pattern in these tumors.

Generally, there are two approaches when characterizing an aggressive pituitary tumor: a histopathological and a clinical one. For the histopathological approach, markers of proliferation, such as a high Ki-67 index, increased mitoses, or extensive p53 staining are considered hallmarks of a more aggressive behavior. This was reflected in the 2004 WHO classification of pituitary adenomas, with Ki-67 labeling index >3%, and extensive p-53 staining as classification criteria for so called atypical adenomas [1]. This combination of proliferation markers is found in 3–15% of all pituitary adenomas [10, 11]. The WHO 2004 classification was in line with observations that the vast majority of pituitary adenomas have Ki-67 indexes <3%, whereas >10% is mainly seen in pituitary tumors with a more aggressive behavior [12]. Similarly, high p53 expression is more often found in aggressive pituitary tumors [12].

The new WHO 2017 classification does not refer to atypical adenomas as distinct entity and only distinguishes pituitary carcinomas [3]. The main reason is twofold. Low Ki-67 and negative p53 staining is found in approximately 20% of true aggressive pituitary tumors and pituitary carcinomas [13], showing that these markers, although related to risk of recurrence [14] will not perfectly predict aggressive adenoma behavior. Also there is no agreement on the set of markers and cut-offs to be used [1]. The 2004 WHO classification of atypical pituitary adenomas was therefore not considered sufficient to guide management of pituitary adenomas.

However, not only markers of proliferation are still considered relevant for aggressive behavior [3], also local factors like cavernous sinus and/or sphenoid sinus invasion and tumor microenvironment, are considered relevant. Secretion of chemokines by the tumor facilitate macrophage, neutrophil, and T cell recruitment into the tumor [15], and tumor associated fibroblasts cultured from clinically non-functioning adenomas and somatotropinomas, secreted more IL-6 in those cases that presented with cavernous sinus invasion compared to fibroblasts from non-invasive tumors [16].

A second approach considers an invasive tumor with abnormal growth pattern and multiple recurrences despite multimodality treatment including surgery, radiotherapy, and medical treatment as indicative for an aggressive pituitary adenoma [2]. Such clinical characterization of aggressiveness leaves room for different interpretations as many aspects are undefined. How many recurrences, over what time period and following what kind of treatments? We likely see an acromegaly patient with a third recurrence in 25 years despite surgery, radiotherapy and medical treatment different from a patient with a fourth biochemical recurrence in two years with clear signs of further growth of the large tumor. This lack of clarity with regard to the definition will thus hamper an accurate estimation of the incidence of aggressive pituitary adenomas. Mind that this definition explicitly takes the invasiveness, as well as the unusual growth pattern into account, meaning that an adenoma that is difficult to treat only from a biochemical perspective is not considered an aggressive pituitary adenoma. Also, it is not only the size of the tumor that determines its aggressiveness, and in giant prolactinomas (tumor diameter > 4 cm) excellent responses to medical therapy are well-known [17]. In a large series of aggressive pituitary adenomas, the majority was clinically functioning [13].

The definition of an aggressive adenoma includes invasiveness; in a large series of nonfunctioning pituitary macroadenomas treated surgically, 94% of patients had residual tumor visible on postoperative imaging [18]; in a pathological series of patients with functioning and nonfunctioning adenomas, microscopic evidence of dural invasion was found in 88% of macroadenomas and 94% of adenomas with extrasellar extension [19]. Mind that these numbers cannot be translated to all pituitary adenomas, even not macroadenomas, as these series are based on surgically treated adenomas, i.e. a selection of all (macro)adenomas with a higher likelihood of invasiveness.

If we consider regrowth after treatment as the first hallmark of aggressiveness, several studies show that approximately 20–50% of patients with a nonfunctioning adenoma display signs of regrowth five years after initial surgery [9, 20]; this number is lower if additional radiotherapy is applied [20]. After ten years, the percentage of patients with a regrowth of the adenoma is >50% [20]. In series on hormone secreting adenomas, the recurrence risk is lower after medical treatment or surgery, but it needs to be acknowledged that in prolactinomas [21], acromegaly [22] and Cushing’s disease [23–25] the tumor size at detection and treatment is smaller compared to nonfunctioning adenomas.

In large a series of 765 patients with a nonfunctioning pituitary adenoma, 90 had a second tumor regrowth; the estimated 5-year probability of second regrowth was 35% after a first regrowth [18]. In the same study, the 5-year probability of a third regrowth was 26%. Importantly, these numbers were dependent on type of treatment, with the lowest regrowth probability after treatment with radiotherapy and/or surgery. For example, the risk for a second regrowth was 13% after combined surgery and radiotherapy, and 63% in patients with an expectant approach. These data suggest that 2–3% of patients with a macroadenoma display a regrowth a least 3 times.

Two important lessons can thus be learned from this study. Firstly, a number of patients harboring a pituitary adenoma show multiple recurrences over time; secondly, this risk is related to treatment modalities. Still, this 2–3% does not represent the percentage of patients with an aggressive adenoma according to the definition outlined above, as many of these patients did not have multiple lines of treatment. We could, however, argue that ~2% reflects the upper limit of the percentage of patients with a pituitary macroadenoma that can be classified as an aggressive adenoma.

In a large database, containing tissues from 3489 pituitary adenomas, 5 (0.12%) were classified as pituitary carcinoma [26]. Mind however that this is not a reflection of the malignancy risk in all pituitary adenomas as this series is a selection, i.e. adenomas for which surgical treatment was considered appropriate. In the largest series published on aggressive pituitary adenomas and pituitary carcinomas, 40 carcinomas and 116 aggressive adenomas were included [13]. Although the sampling frame of the study was not well-defined, it suggests that an aggressive adenoma is 3–4 times more common than a pituitary carcinoma. This would translate in an estimated percentage of 0.3–0.5% of all macroadenomas being aggressive.

There is currently a debate whether aggressive pituitary adenomas and carcinomas are categories that need to be separated [27]. And the more general question is whether indolent adenomas that escape detection, pituitary adenomas with a course that comes to clinical attention, and aggressive pituitary adenomas/carcinomas comprise fully distinct entities. Currently, there are no convincing arguments that these are biologically and clinically distinct entities; more likely the categories overlap and adenomas can even change their behavior during life [28, 29]. This is depicted in Fig. 1.

To conclude, less than 0.1% of all detected pituitary adenomas will become malignant, and probably around 0.5% will display an aggressive course. If we consider all adenomas (also the small adenomas with an indolent course), the percentage aggressive pituitary adenomas/carcinomas is much lower (see Fig. 1). However, the exact incidence of both aggressive pituitary adenomas and pituitary carcinomas is unknown. This is related to two factors:

Most data come from series with selected patients, such as surgically treated patients, which is likely not a reflection of all patients with a pituitary adenoma. The reported and estimated incidence of aggressive behavior (~0.5%) and pituitary carcinoma (~0.1) is thus a likely overestimation of the true risk.

An aggressive pituitary adenoma is not well-defined; even though an overarching definition, capturing both immunohistochemical and clinical characteristics is probably not waterproof, adoption of a widely accepted definition will be very helpful to harmonize research and establish more reliable epidemiological data.
